# Clinical features of Japanese patients with acute hepatic porphyria

**DOI:** 10.1002/jmd2.12336

**Published:** 2022-10-13

**Authors:** Yutaka Horie, Yuka Yasuoka, Tomohide Adachi

**Affiliations:** ^1^ Department of Gastroenterology Saiseikai Gotsu General Hospital Gotsu Japan; ^2^ Medical Affairs Alnylam Japan KK Tokyo Japan; ^3^ Department of General Medicine and Neurology Saiseikai Central Hospital Tokyo Japan

**Keywords:** claims, Japan, porphyria, prevalence

## Abstract

Acute hepatic porphyria (AHP) is a family of rare genetic diseases of heme biosynthesis characterized by severe neurovisceral attacks. The clinical characteristics of patients with AHP as well as the prevalence of AHP in Japan are not well understood. The objectives of this study were to describe clinical characteristics of AHP at time of diagnosis in Japanese patients and to estimate the prevalence of AHP. Patients with porphyria were selected from Japan's Medical Data Vision health care claims database between April 2008 and June 2020. Patient characteristics before and at time of AHP diagnosis were evaluated. Prevalence per 100 000 was estimated during the study period. A total of 391 cases of AHP were included. At time of AHP diagnosis, mean age was 44 years, and the most common type was acute intermittent porphyria. Median time to diagnosis was 3 months, but some patients remained undiagnosed for several years. The most common complications included metabolic disorders (54%) and diabetes mellitus (39%). In addition, the well‐known complications of AHP, including hypertension (22%) and malignant neoplasms of digestive organs (22%), were observed. About 10% of patients received medications that may have aggravated porphyria attacks. The estimated prevalence of AHP in Japan during the study period was 1.18 cases per 100 000 population. At time of diagnosis, many patients with AHP in Japan are already experiencing a high burden of disease‐related complications. Raising AHP awareness may aid physicians in providing an earlier diagnosis and reducing lifetime disease burden.

**Synopsis**

Disease‐related complications are present at the time of acute hepatic porphyria diagnosis, highlighting the need for awareness and early diagnosis of this rare disease.

## 1 INTRODUCTION

Acute hepatic porphyria (AHP) is a rare and potentially life‐threatening inherited disease caused by genetic mutations in the heme biosynthetic enzymes of the liver.^1^ There are four types of AHP: autosomal‐dominant acute intermittent porphyria (AIP), hereditary coproporphyria (HCP), variegate porphyria (VP), and the extremely rare autosomal‐recessive delta‐aminolevulinic acid (ALA) dehydratase deficient porphyria. AHP presents with a wide variety of nonspecific symptoms and common disorders (e.g., abdominal pain, anxiety, liver complications), often resulting in a succession of treatments by a variety of physician specialists and missed or delayed diagnoses.^2^

One study of a consortium of patients with AHP in the United States (*N* = 108) found that average time from onset of symptoms to definitive diagnosis was 15 years, which suggests that patient outcomes may greatly improve with earlier detection.^3^ Large national health care claims databases now allow for investigators to gain insights into how diseases are diagnosed and treated within routine clinical practice. This information is increasingly important, particularly for rare diseases, as real‐world evidence regarding diagnosis and treatment practices is needed to increase awareness and enable earlier diagnosis and treatment of conditions that are challenging to recognize.

The small number of documented cases of rare diseases such as AHP, along with the commonality of symptoms, also makes it difficult to precisely estimate the prevalence of AHP worldwide. Among the four types of AHP, AIP is the most common, with a minimum estimated prevalence of disease‐related mutations of ~1 in 1299.^4^ Penetrance is estimated to be about 23% in families with AIP, but only 0.5%–1% in the general population.^1^, ^4^ Additionally, in Europe, the prevalence of symptomatic AHP overall is an estimated 0.5 case per 100 000 population.^5^ Further research is needed to add evidence and detail to published aggregate estimates of AHP prevalence.

The main objective of this study was to describe clinical characteristics of AHP at time of diagnosis—using data from patients with AHP in a large health care database in Japan—to better understand the clinical profile of patients with AHP at diagnosis and to aid physicians in identifying patients with this disease. Estimation of the prevalence of AHP within the database population was a secondary objective.

## 2 METHODS

This study used the Medical Data Vision (MDV) health care claims database, which covers ~24% of acute‐care hospitals in Japan and has more than 1 million health care claims entered each month. All data were fully deidentified and anonymous. The MDV database does not link data between hospitals; therefore, it is possible that patients may have received diagnoses and treatments outside of what is recorded in the database. The MDV database began collecting claims data in April 2008, and, therefore, cases registered with a diagnosis of porphyria were identified using *ICD‐10* (*International Classification of Diseases, Tenth Revision*) codes between April 2008 and June 2020. Porphyria types identified as AHP included AIP, HCP, VP, acute porphyria (AP), and hepatic porphyria (HP). AP and HP are not included among the four accepted, distinguishable types of AHP; therefore, the clinical characteristics individually analyzed were restricted to the AIP, HCP, and VP types. Patients with hereditary erythropoietic porphyria, erythropoietic porphyria, protoporphyria, porphyria cutanea tarda, porphyria, and congenital porphyria were excluded. For patients with multiple diagnoses of porphyria, only the latest diagnosis was counted toward the overall sample.

AHP diagnostic criteria, developed by the Japan Intractable Diseases Information Center, use clinical, laboratory, or genetic testing to make an AHP diagnosis.^6^ In the MDV database, each patient's diagnosis status was documented with a flag indicating “suspected” or “confirmed,” which was determined by a physician. The MDV database does not include genetic testing data because genetic testing was not covered by national insurance until April 2022, and laboratory data were not available for all cases, as these data were collected only from a limited number of institutions.^7^ Therefore, both confirmed and suspected cases were included as AHP cases in this study.

Patient demographics and clinical characteristics at time of and leading up to diagnosis were tabulated; these data included age, sex, age at first visit to health care facility, and time to diagnosis. Time of diagnosis was defined as the month when AHP was first diagnosed, and time to diagnosis was defined as the time between first visit to the health care facility and time of diagnosis. In addition, physician department, complications, and concomitant medication use that may have aggravated AHP attacks were also tabulated. *ICD‐10* codes were also used to document complications. For example, liver disease was tabulated using diagnoses with *ICD‐10* codes K70–K77, which included alcoholic, toxic, and other inflammatory liver diseases; hepatic failure; chronic hepatitis; fibrosis and cirrhosis of the liver; and other liver disorders in diseases classified elsewhere.

Prevalence of AHP was evaluated over the entire study period. The number of recorded AHP cases was divided by the number of patients in the MDV database during the same period and was reported per 100 000 population. A similar approach has been taken in other studies using the MDV database in Japan.^8^

## 3 RESULTS

**FIGURE 1:**
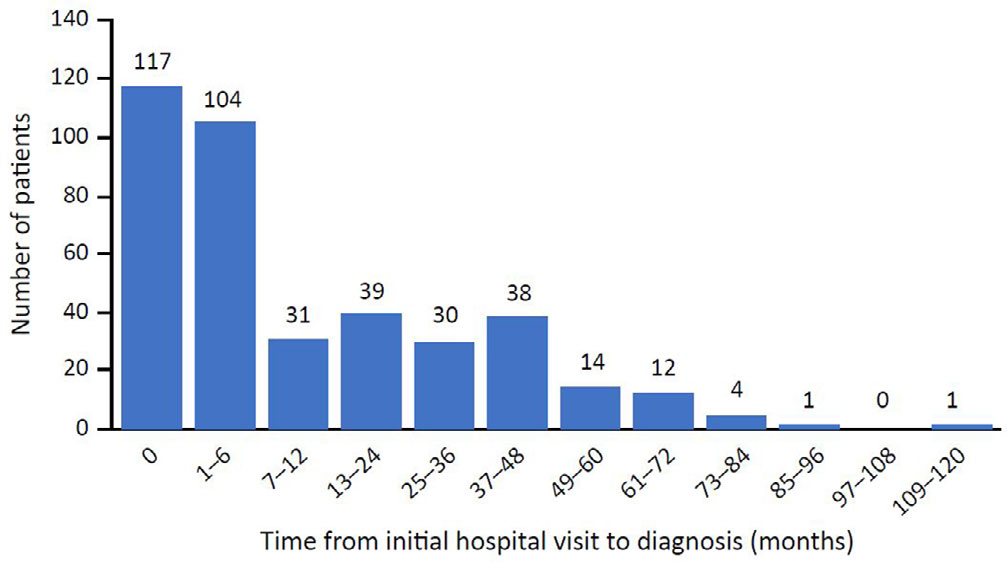
Distribution of time from initial hospital visit to AHP diagnosis. AHP, acute hepatic porphyria

In the MDV database, 1430 registered cases of porphyria were identified, including 391 registered cases of AHP. Of the registered AHP cases, 120 were AIP, 46 were HCP, and 11 were VP (Table 1). The mean age at AHP diagnosis was 44 years, and 55% of patients were female (Table 1). On average, patients with HCP were younger than patients with other types of porphyria. For patients with AHP, the median time to diagnosis at the facility was 3 months. Although most patients were diagnosed within 3 months of their first visit, some patients remained undiagnosed for more than 4 years (Table 1 and Figure 1).

**TABLE 1:** Patient demographics at time of AHP diagnosis

	Overall	Type		
		AIP	HCP	VP
Cases, *n* ^a^	391	120	46	11
Sex, *n* (%)				
Male	177 (45.3)	43 (35.8)	27 (58.7)	6 (54.5)
Female	214 (54.7)	77 (64.2)	19 (41.3)	5 (45.5)
Age, years				
Mean (SD)	44.4 (23.1)	42.6 (19.0)	34.4 (27.1)	56.1 (15.1)
Median	44.0	39.5	25.5	57.0
Q1–Q3	26.0–64.0	29.0–55.5	11.0–60.0	41.0–68.0
Min–max	0–94	11–94	0–88	31–73
Time to diagnosis, months				
Median	3.0	1.0	2.0	1.0
Q1–Q3	0.0–26.0	0.0–16.5	0.0–19.0	0.0–15.0
Min–max	0–111	0–64	0–80	0–33
Estimated prevalence per 100 000 population	1.18	0.36	0.14	0.03

Abbreviations: AHP, acute hepatic porphyria; AIP, acute intermittent porphyria; HCP, hereditary coproporphyria; max, maximum; min, minimum; Q, quintile; SD, standard deviation; VP, variegate porphyria.

^a^ Cases included both suspected and confirmed cases of AHP.

Patients were most often diagnosed within departments of internal medicine (35%), dermatology (23%), and neurology (16%; Table SS1). AIP was often diagnosed within a neurology department, and HCP within a dermatology department.

The most common complications at AHP diagnosis were metabolic disorders, diabetes mellitus, and esophageal, gastric, and duodenal diseases (Table 2 lists complications with incidence of 20% or greater). In the MDV database general population, 22% were diagnosed with diabetes mellitus, compared with 39% of the AHP population. Symptoms and signs related to the digestive system and abdomen occurred more frequently in patients with AIP, whereas dermatologic conditions (dermatitis and eczema) occurred more frequently in patients with HCP and less frequently in patients with AIP. In addition, some well‐known complications of AHP, including hypertension (22%) and malignant neoplasms of digestive organs (22%), were seen at diagnosis. Of the 84 cases of malignant neoplasms of digestive organs observed, the most frequent were neoplasms of the colon (*n* = 35), liver and intrahepatic bile ducts (*n* = 29), stomach (*n* = 27), and pancreas (*n* = 21). In the MDV database general population, 15% were diagnosed with malignant neoplasms of digestive organs, compared with 22% of the AHP population. Specifically, regarding malignant neoplasms of the liver and intrahepatic bile ducts, the frequency observed in the AHP population was more than double that of the MDV database general population (7% vs. 3%, respectively). Conversely, kidney‐related complications occurred less frequently (<20%) at diagnosis. Medications that were being taken at time of diagnosis and that may have aggravated porphyria attacks included some barbiturates, estrogens, and antihistamines (Table 3).^9^ During the study period, the MDV database included 33 161 332 individuals, yielding an overall AHP prevalence of 1.18 per 100 000 population. Prevalence of AIP, HCP, and VP was 0.36, 0.14, and 0.03 per 100 000 population, respectively (Table 1).

**TABLE 2:** Complications at time of AHP diagnosis, *n* (%)^a^

	Overall	Type		
		AIP	HCP	VP
Cases, *n* ^b^	391	120	46	11
Complications (ICD‐10), *n* (%)				
Metabolic disorders (E70–E90)	209 (53.5)	62 (51.7)	26 (56.5)	5 (45.5)
Diabetes mellitus (E10–E14)	153 (39.1)	49 (40.8)	13 (28.3)	5 (45.5)
Diseases of esophagus, stomach, and duodenum (K20–K31)	152 (38.9)	53 (44.2)	15 (32.6)	4 (36.4)
Systemic connective tissue disorders (M30–M36)	134 (34.3)	36 (30.0)	22 (47.8)	4 (36.4)
Disorders of thyroid gland (E00–E07)	129 (33.0)	46 (38.3)	7 (15.2)	4 (36.4)
Other diseases of intestines (K55–K64)	118 (30.2)	37 (30.8)	5 (10.9)	4 (36.4)
Dermatitis and eczema (L20–L30)	115 (29.4)	21 (17.5)	26 (56.5)	2 (18.2)
Episodic and paroxysmal disorders (G40–G47)	109 (27.9)	40 (33.3)	7 (15.2)	1 (9.1)
Diseases of liver (K70–K77)	108 (27.6)	26 (21.7)	6 (13.0)	3 (27.3)
Symptoms and signs involving digestive system and abdomen (R10–R19)	102 (26.1)	44 (36.7)	6 (13.0)	2 (18.2)
Nutritional anemias (D50–D53)	92 (23.5)	24 (20.0)	11 (23.9)	2 (18.2)
Viral hepatitis (B15–B19)	88 (22.5)	21 (17.5)	7 (15.2)	3 (27.3)
Hypertensive diseases (I10–I15)	86 (22.0)	22 (18.3)	8 (17.4)	1 (9.1)
Malignant neoplasms of digestive organs (C15–C26)	84 (21.5)	26 (21.7)	6 (13.0)	3 (27.3)
Malignant neoplasm of esophagus	2 (0.5)	1 (0.8)	0 (0.0)	1 (9.1)
Malignant neoplasm of stomach	27 (6.9)	11 (9.2)	3 (6.5)	0 (0.0)
Malignant neoplasm of small intestine	4 (1.0)	2 (1.7)	0 (0.0)	0 (0.0)
Malignant neoplasm of colon	35 (9.0)	12 (10.0)	4 (8.7)	1 (9.1)
Malignant neoplasm of rectosigmoid junction	1 (0.3)	0 (0.0)	0 (0.0)	0 (0.0)
Malignant neoplasm of rectum	3 (0.8)	1 (0.8)	0 (0.0)	1 (9.1)
Malignant neoplasm of liver and intrahepatic bile ducts	29 (7.4)	2 (1.7)	3 (6.5)	0 (0.0)
Malignant neoplasm of pancreas	21 (5.4)	8 (6.7)	0 (0.0)	2 (18.2)
General symptoms and signs (R50–R69)	82 (21.0)	28 (23.3)	10 (21.7)	0 (0.0)
Other forms of heart disease (I30–I52)	81 (20.7)	31 (25.8)	6 (13.0)	2 (18.2)
Other nutritional deficiencies (E50–E64)	80 (20.5)	33 (27.5)	4 (8.7)	3 (27.3)

Abbreviation: AHP, acute hepatic porphyria; AIP, acute intermittent porphyria; HCP, hereditary coproporphyria; ICD‐10, International Classification of Diseases, Tenth Revision; VP, variegate porphyria.

^a^ Complications with incidence of 20% or greater are shown. Suspected and/or confirmed cases of acute porphyria and hepatic porphyria not shown in table.

^b^ Cases included both suspected and confirmed cases of AHP.

**TABLE 3:** Concomitant medications that may have aggravated acute AHP attacks

	Overall	Type		
		AIP	HCP	VP
Cases, *n* ^a^	391	120	46	11
Medication at diagnosis (*n*, %)				
Butylscopolamine bromide	38 (9.7)	19 (15.8)	2 (4.3)	2 (18.2)
Lidocaine hydrochloride	33 (8.4)	15 (12.5)	2 (4.3)	3 (27.3)
Hydroxyzine hydrochloride	28 (7.2)	10 (8.3)	1 (2.2)	0 (0.0)
Metoclopramide	26 (6.6)	12 (10.0)	1 (2.2)	0 (0.0)
Spironolactone	9 (2.3)	3 (2.5)	0 (0.0)	1 (9.1)
Sodium valproate	8 (2.0)	2 (1.7)	2 (4.3)	0 (0.0)
Lidocaine	7 (1.8)	3 (2.5)	0 (0.0)	0 (0.0)
Carbamazepine	6 (1.5)	1 (0.8)	1 (2.2)	0 (0.0)
Lidocaine hydrochloride and adrenaline	6 (1.5)	2 (1.7)	0 (0.0)	1 (9.1)
Sulfamethoxazole and trimethoprim	5 (1.3)	1 (0.8)	0 (0.0)	0 (0.0)
Glimepiride	4 (1.0)	0 (0.0)	0 (0.0)	0 (0.0)
Phenytoin sodium	4 (1.0)	3 (2.5)	0 (0.0)	0 (0.0)
Mepivacaine hydrochloride	4 (1.0)	3 (2.5)	1 (2.2)	0 (0.0)
Tramadol hydrochloride	3 (0.8)	2 (1.7)	0 (0.0)	0 (0.0)
Nifedipine	3 (0.8)	1 (0.8)	0 (0.0)	0 (0.0)
Dydrogesterone	2 (0.5)	2 (1.7)	0 (0.0)	0 (0.0)
Tramadol hydrochloride and acetaminophen	2 (0.5)	0 (0.0)	0 (0.0)	0 (0.0)
Phenobarbital	2 (0.5)	0 (0.0)	0 (0.0)	0 (0.0)
Erythromycin ethylsuccinate	1 (0.3)	0 (0.0)	1 (2.2)	0 (0.0)
Erythromycin stearate	1 (0.3)	1 (0.8)	0 (0.0)	0 (0.0)
Clindamycin phosphate	1 (0.3)	0 (0.0)	1 (2.2)	0 (0.0)
Glibenclamide	1 (0.3)	0 (0.0)	0 (0.0)	0 (0.0)
Triclofos sodium	1 (0.3)	0 (0.0)	0 (0.0)	0 (0.0)
Phenytoin	1 (0.3)	0 (0.0)	0 (0.0)	0 (0.0)
Ranitidine hydrochloride	1 (0.3)	0 (0.0)	0 (0.0)	0 (0.0)
Lamotrigine	1 (0.3)	1 (0.8)	0 (0.0)	0 (0.0)

Abbreviation: AHP, acute hepatic porphyria; AIP, acute intermittent porphyria; HCP, hereditary coproporphyria; VP, variegate porphyria.

^a^ Cases included both suspected and confirmed cases of AHP.

## 4 DISCUSSION

This study is the first to describe clinical characteristics preceding and at time of diagnosis among patients with AHP in Japan. The study findings regarding complications occurring proximal to AHP diagnosis may have important implications in terms of disease management. Understanding which patient characteristics and symptoms are most common at time of diagnosis may help physicians identify patients with AHP in routine clinical practice, across multiple departments and specialties. In this study, for example, metabolic disturbances in AHP were frequent, as were gastrointestinal symptoms at time of diagnosis. Dermatologic conditions were observed in approximately one‐third of patients with AHP, and were most common in patients with HCP. While dermatologic manifestations are rare in AHP, they can occur in AHP types, particularly VP and HCP.^10^

Additionally, a high rate of diabetes was found in patients with AHP. No prior research has documented a high incidence rate of diabetes in this patient population, but several factors may contribute to the complication. Results from a recently reported observational case‐control study documented a high rate of insulin resistance in a population of 44 patients with AIP, suggesting a possible biological mechanism for the high rate of diabetes observed in these patients.^11^ It is also possible that racial biological differences contribute to the increased incidence rate. Some investigators have postulated that global migration has led to ethnic differences in insulin sensitivity, and these differences influence the risk of diabetes. Such a difference accounts for Asians being more and differentially susceptible to diabetes compared with Caucasians.^12^

It is widely reported that the prevalence and incidence of chronic kidney disease, hypertension, and hepatocellular carcinoma are increased in patients with AHP because of the long‐term toxicity associated with ALA.^13^, ^14^ In this study, liver disease (28%), hypertension (22%), and malignant neoplasms of digestive organs (22%) all had an incidence of 20% or greater at the time of diagnosis. However, kidney‐related complications had a lower incidence at the time of diagnosis, suggesting that hypertension and hepatic disease may occur earlier than kidney dysfunction in this patient population. Further investigation into the development of complications after diagnosis is needed.

Additional research is required to fully understand the potential implications of some findings of this study. For example, AHP is known to occur after puberty, when menstruation begins, but this database study found an average age at diagnosis of 44 years, similar to the delayed diagnosis seen in US patients.^3^ Reasons for delayed diagnosis may be the lack of characteristic symptoms associated with AHP, the difficulty in diagnosing this rare disease, and the transience of severe symptoms. Additionally, as the MDV database does not link data between hospitals, it is possible some patients were previously diagnosed elsewhere. Within AHP, age at diagnosis was earlier in patients with HCP than for the other porphyria types, but the reason for this is unclear. The percentage of female patients with AHP was lower in this sample (64%, 41%, and 46% for AIP, HCP, and VP, respectively) compared with other findings. In a US porphyria consortium study, females made up 83%, 77%, and 66% of AIP, HCP, and VP populations, respectively.^3^ The reason for the lower proportion in this study is unclear. However, one possible reason is that, since genetic testing was not covered by national insurance, the diagnosis was being made without genetic testing.

Finally, this study yielded an estimated AIP prevalence of 0.36 case per 100 000 population, consistent with other estimates in the literature. Prior genealogical research recorded the number of genetically confirmed porphyria cases in the San‐in region (population 1.3 million) of Japan over a 40‐year period (1974–2014).^1^, ^15^ That research documented five families with AIP, one family with VP, and four families with erythropoietic protoporphyria in the region. Assuming two or three patients and carriers per family based on prior literature,^4^ the total number of porphyria cases among the San‐in region's 1.3 million inhabitants was estimated to be 2 per 100 000 population, including 1 case of AIP per 100 000.^15^

While the genealogical research in the San‐in region of Japan estimated 1 case of AIP per 100 000 population, the present database study estimated a prevalence of 0.36 per 100 000. Considering the penetration rate in families with a known inheritance of AIP is estimated at 23%, and that of the general population is estimated at 0.5%–1%, the number of AIP cases seen in this database study may be similar to that of the general population.^4^

Ratios of porphyria types have also been studied previously. In Europe, the incidence ratios of symptomatic cases of AIP, HCP, and VP were estimated to be 1.00, 0.15, and 0.62, respectively.^5^ In this study, these ratios were 1.00, 0.38, and 0.09, respectively. The proportion of symptomatic cases among patients with VP was lower in this study than in Europe. Furthermore, according to a literature review that summarized cases reported in Japan over a 91‐year period (1920–2010),^16^ these ratios were 1.00, 0.20, and 0.29, respectively—demonstrating a trend similar to that of the present data and suggesting the VP rate is lower in Japan than in Europe.

This study has several strengths. The results provide clinical evidence from a single cohort of Japanese patients, which is important in understanding the impact of AHP on patients with varying demographics worldwide. The MDV database provides a large representative sample of anonymized Japanese patient data, medical costs, and laboratory results.^7^ Additionally, information regarding clinical evaluation of AHP and its types is limited in the literature. This study provides a clinical description of patients who are experiencing several types of porphyria across multiple medical specialties and/or departments, which may assist physicians in recognizing these cases earlier in the disease progression.

Despite its strengths, this study has several limitations as well. As noted earlier, the MDV database does not link data between hospitals, so it is possible some patients may have received their diagnoses and treatments outside of what is recorded in the database. In addition, not all laboratory data are captured in the MDV database. Furthermore, because genetic testing for AHP was not covered by insurance in Japan, genetic confirmation of the AHP diagnosis could not be directly ascertained through the MDV database. However, AHP diagnostic criteria in Japan recommend that diagnosis be based on clinical, laboratory, or genetic testing data.^6^ Therefore, the confirmed cases of AHP could possibly correlate to indirect laboratory or genetic testing confirmations. Regardless of the limitations inherent to the use of administrative data, such databases are particularly useful in studying rare diseases, such as AHP, for which clinical research studies are often limited by small sample sizes and missing data. It is important to report these results to contribute to the limited AHP data currently available and determine areas for additional research. Further research should be conducted to identify the demographic and clinical characteristics of patients with AHP upon genetic confirmation.

## 5 CONCLUSIONS

This analysis of a large‐scale national health care claims database in Japan found that, at time of diagnosis, patients with AHP often experience significant clinical burden in terms of complications. The results demonstrate an urgent need to diagnose AHP early to reduce complications and limit use of concomitant medications that may have an adverse impact on patients with AHP. As the median time from first hospitalization to diagnosis was observed to be short, raising AHP awareness may help patients receive an earlier diagnosis and reduce their lifetime disease burden.

## AUTHOR CONTRIBUTIONS

Conceptualization, methodology, and software: Yutaka Horie, Yuka Yasuoka, and Tomohide Adachi.

Data curation and writing—original draft preparation: Yutaka Horie, Yuka Yasuoka, and Tomohide Adachi (Note: Data curation was by Medical Data Vision).

Visualization and investigation: Yutaka Horie, Yuka Yasuoka, and Tomohide Adachi.

Supervision: Yutaka Horie, Yuka Yasuoka, and Tomohide Adachi.

Software and validation: No special software was developed or validated for this study; all analyses were performed using the Medical Data Vision database by Intage Healthcare Inc., using Microsoft Excel, and under the direction and oversight of the study authors.

Writing—reviewing and editing: Yutaka Horie, Yuka Yasuoka, and Tomohide Adachi. Guarantor: Yuka Yasuoka.

## CONFLICT OF INTEREST

Yutaka Horie reported receiving consultant fees from Alnylam Pharmaceuticals. Yuka Yasuoka reported being employed by and owning stock and stock options in Alnylam Japan KK. Tomohide Adachi has nothing to disclose.

## ETHICS STATEMENT

Not applicable; database study using deidentified and anonymous data.

## PATIENT CONSENT STATEMENT

Not applicable; database study using deidentified and anonymous data.

## Supporting information


**TABLE S1** Departments visited at time of diagnosis^a^
**TABLE S2** Concomitant medications that may have aggravated acute AHP attacks

## Data Availability

Not applicable; database study using deidentified and anonymous data.
