# Supplementary material for: Clinical features of Japanese patients with acute hepatic porphyria
Source: JIMD Rep. 2022 Oct 13;64(1):71–8. doi: 10.1002/jmd2.12336 (PMC9830008; doi:10.1002/jmd2.12336)
Supplement: Supplementary file 1 — TABLE S1 Departments visited at time of diagnosisa TABLE S2 Concomitant medications that may have aggravated acute AHP attacks [file JMD2-64-71-s001.docx]

**SUPPLEMENTAL MATERIAL FOR:**

**Clinical Features of Japanese Patients with Acute Hepatic Porphyria**

Yutaka Horie^a^; Yuka Yasuoka^b^; Tomohide Adachi^c^

^a^Department of Gastroenterology, Saiseikai Gotsu General Hospital, Shimane, Japan;
^b^Alnylam Japan KK, Tokyo, Japan; ^c^Department of General Medicine and Neurology,
Saiseikai Central Hospital, Tokyo, Japan

## **SUPPLEMENTAL TABLE 1** Departments visited at time of diagnosis^a^

|  | **Overall** | **Subtype** | | |
| --- | --- | --- | --- | --- |
|  |  | **Acute intermittent porphyria** | **Hereditary coproporphyria** | **Variegate porphyria** |
| **Cases, n^b^** | 391 | 120 | 46 | 11 |
| **Department at diagnosis, n (%)** |  |  |  |  |
| Internal medicine | 138 (35.3) | 44 (36.7) | 10 (21.7) | 1 (9.1) |
| Dermatology | 88 (22.5) | 8 (6.7) | 27 (58.7) | 3 (27.3) |
| Neurology | 63 (16.1) | 42 (35.0) | 3 (6.5) | 0 (0.0) |
| Gastroenterology | 37 (9.5) | 9 (7.5) | 2 (4.3) | 3 (27.3) |
| Pediatrics | 36 (9.2) | 3 (2.5) | 6 (13.0) | 0 (0.0) |
| Gastroenterology | 29 (7.4) | 5 (4.2) | 1 (2.2) | 0 (0.0) |
| Obstetrics and gynecology | 24 (6.1) | 9 (7.5) | 0 (0.0) | 0 (0.0) |
| Psychiatry | 21 (5.4) | 5 (4.2) | 0 (0.0) | 1 (9.1) |
| Orthopedics | 19 (4.9) | 8 (6.7) | 3 (6.5) | 1 (9.1) |
| Ophthalmology | 19 (4.9) | 7 (5.8) | 2 (4.3) | 0 (0.0) |
| Surgery | 13 (3.3) | 3 (2.5) | 2 (4.3) | 2 (18.2) |
| Otolaryngology | 13 (3.3) | 3 (2.5) | 2 (4.3) | 0 (0.0) |
| Neurosurgery | 12 (3.1) | 5 (4.2) | 2 (4.3) | 0 (0.0) |
| Urology | 12 (3.1) | 5 (4.2) | 0 (0.0) | 0 (0.0) |
| General medicine | 10 (2.6) | 7 (5.8) | 1 (2.2) | 0 (0.0) |
| Endocrinology | 9 (2.3) | 3 (2.5) | 0 (0.0) | 3 (27.3) |
| Emergency medicine | 9 (2.3) | 6 (5.0) | 0 (0.0) | 0 (0.0) |
| Circulatory medicine | 8 (2.0) | 6 (5.0) | 0 (0.0) | 0 (0.0) |
| Respiratory medicine | 6 (1.5) | 5 (4.2) | 0 (0.0) | 0 (0.0) |
| Hematology | 6 (1.5) | 2 (1.7) | 0 (0.0) | 0 (0.0) |

^a^Departments visited at time of emergency admissions were tabulated. Multiple (≥1) departments visited within same month of diagnosis were counted separately. Data tabulated for period from first to last visit.
^b^Cases included both suspected and confirmed cases of acute hepatic porphyria.

**SUPPLEMENTAL TABLE 2** Concomitant medications that may have aggravated acute AHP attacks

|  | Overall | Subtype | | |
| --- | --- | --- | --- | --- |
|  |  | **Acute intermittent porphyria** | **Hereditary coproporphyria** | **Variegate porphyria** |
| Cases, n^a^ | 391 | 120 | 46 | 11 |
| Medication at diagnosis (n, %) |  |  |  |  |
| Butylscopolamine bromide | 38 (9.7) | 19 (15.8) | 2 (4.3) | 2 (18.2) |
| Lidocaine hydrochloride | 33 (8.4) | 15 (12.5) | 2 (4.3) | 3 (27.3) |
| Hydroxyzine hydrochloride | 28 (7.2) | 10 (8.3) | 1 (2.2) | 0 (0.0) |
| Metoclopramide | 26 (6.6) | 12 (10.0) | 1 (2.2) | 0 (0.0) |
| Spironolactone | 9 (2.3) | 3 (2.5) | 0 (0.0) | 1 (9.1) |
| Sodium valproate | 8 (2.0) | 2 (1.7) | 2 (4.3) | 0 (0.0) |
| Lidocaine | 7 (1.8) | 3 (2.5) | 0 (0.0) | 0 (0.0) |
| Carbamazepine | 6 (1.5) | 1 (0.8) | 1 (2.2) | 0 (0.0) |
| Lidocaine hydrochloride and adrenaline | 6 (1.5) | 2 (1.7) | 0 (0.0) | 1 (9.1) |
| Sulfamethoxazole and trimethoprim | 5 (1.3) | 1 (0.8) | 0 (0.0) | 0 (0.0) |
| Glimepiride | 4 (1.0) | 0 (0.0) | 0 (0.0) | 0 (0.0) |
| Phenytoin sodium | 4 (1.0) | 3 (2.5) | 0 (0.0) | 0 (0.0) |
| Mepivacaine hydrochloride | 4 (1.0) | 3 (2.5) | 1 (2.2) | 0 (0.0) |
| Tramadol hydrochloride | 3 (0.8) | 2 (1.7) | 0 (0.0) | 0 (0.0) |
| Nifedipine | 3 (0.8) | 1 (0.8) | 0 (0.0) | 0 (0.0) |
| Dydrogesterone | 2 (0.5) | 2 (1.7) | 0 (0.0) | 0 (0.0) |
| Tramadol hydrochloride and acetaminophen | 2 (0.5) | 0 (0.0) | 0 (0.0) | 0 (0.0) |
| Phenobarbital | 2 (0.5) | 0 (0.0) | 0 (0.0) | 0 (0.0) |
| Erythromycin ethylsuccinate | 1 (0.3) | 0 (0.0) | 1 (2.2) | 0 (0.0) |
| Erythromycin stearate | 1 (0.3) | 1 (0.8) | 0 (0.0) | 0 (0.0) |
| Clindamycin phosphate | 1 (0.3) | 0 (0.0) | 1 (2.2) | 0 (0.0) |
| Glibenclamide | 1 (0.3) | 0 (0.0) | 0 (0.0) | 0 (0.0) |
| Triclofos sodium | 1 (0.3) | 0 (0.0) | 0 (0.0) | 0 (0.0) |
| Phenytoin | 1 (0.3) | 0 (0.0) | 0 (0.0) | 0 (0.0) |
| Ranitidine hydrochloride | 1 (0.3) | 0 (0.0) | 0 (0.0) | 0 (0.0) |
| Lamotrigine | 1 (0.3) | 1 (0.8) | 0 (0.0) | 0 (0.0) |

^a^Cases included both suspected and confirmed cases of AHP.

Abbreviation: AHP, acute hepatic porphyria.
